# Risk of SARS-CoV-2 infection and disease in metastatic triple-negative breast cancer patients treated with immune checkpoint inhibitors

**DOI:** 10.2217/imt-2020-0142

**Published:** 2020-06-03

**Authors:** Patrizia Vici, Laura Pizzuti, Eriseld Krasniqi, Andrea Botticelli, Gennaro Ciliberto, Maddalena Barba

**Affiliations:** ^1^Division of Medical Oncology 2, IRCCS Regina Elena National Cancer Institute, Rome, Italy; ^2^Department of Clinical & Molecular Oncology, University of Rome “Sapienza”, Rome, Italy; ^3^Scientific Direction, IRCCS Regina Elena National Cancer Institute, Rome, Italy

**Keywords:** Covid-19, immunotherapy, metastatic triple-negative breast cancer

Metastatic triple-negative breast cancer (mTNBC) is an aggressive disease with particularly poor outcomes [1]. Over the past few years, relevant gains in knowledge concerning the molecular landscape of this disease have allowed to considerably broaden the available therapeutic armamentarium. Poly ADP-ribose polymerase-1 inhibitors, epigenetic agents, anti-androgens, tyrosine kinase inhibitors and immune checkpoint inhibitors (ICIs) may all optimally exemplify the targeted therapeutic weapons recently gained in the fight against mTNBC [2].

Targeting of immune checkpoints through their respective monoclonal antibodies translates into effective antitumor responses not only in widely recognized ‘immunogenic’ tumor types, for example, melanoma and renal cell carcinoma, but also in other solid tumors including breast cancer [3]. PD-1 is an immune checkpoint expressed on the surface of B cells, T cells and natural killer T cells, with a critical role in modulating self tolerance, immune homeostasis and inflammation. When activated by PD-L1 or -L2, PD-1 mediates downregulation of T-cell activity, causes T-cell lysis and reduces cytokine production significantly [4–8].

In mTNBC, the generally high mutational rates provide a plausible rationale for neoantigen generation and susceptibility to an immunotherapic approach. In addition, TNBC tumors present with more consistent amounts of tumor-infiltranting lymphocytes (TILs) and higher expression of PD-L1 and PD-1 compared with hormone receptor positive breast cancer. These three factors along with the encouraging results from studies of ICIs use in cancers other than breast, fuel great expectations from this therapeutic approach in TNBC [9–13].

Among the currently available ICIs, atezolizumab, which selectively targets PD-L1, was initially approved as single agent for treatment of advanced tumors other than breast cancer. In the IMpassion 130 (NCT02425891) Phase III randomized trial, atezolizumab was then tested, in combination with nab-paclitaxel, as firstline in mTNBC patients. 902 patients were enrolled in this study. Median progression-free survival was 7.2 versus 5.5 months in the atezolizumab plus nab-paclitaxel and in the placebo plus nab-paclitaxel arm, respectively (p = 0.002). The median progression-free survival advantage conferred by atezolizumab was confirmed in the PD-L1-positive subgroup (7.5 vs 5.0 months, respectively; p < 0.0001). At the first interim analysis, there was also a favorable trend in overall survival (OS) (21.3 vs 17.6 months; p = 0.08), which became significant in the PD-L1-positive subgroup in favor of the experimental arm (25.0 vs 15.5 months) [14]. Updated results from intention-to-treat analysis showed a median OS (mOS) of 21.0 versus 18.7 months in the atezolizumab versus placebo arm, respectively (p = 0.078). In the PD-L1-positive subgroup, mOS was 25.0 versus 18.0 months in the placebo arm (hazard ratio: 0.71; 0.54–0.94). On the basis of the above reported results, the US FDA, EMA and Italian Drugs Agency approved atezolizumab in combination with nab-paclitaxel for patients with PD-L1-positive mTNBC [15–17]. At the time of writing, the combination is still not reimbursable in Italy. The results of additional ongoing trials of ICIs combined with chemotherapy are awaited, along with the identification of predictive biomarkers [18]. At present, treatment with a taxane and an ICI represents a remarkable therapeutic option as firstline for mTNBC patients, particularly if taking into account the paucity of effective treatment strategies currently available for patients diagnosed with this prognostically unfavorable breast cancer subtype.

On 12 March 2020, the WHO announced Covid-19 outbreak a pandemic [19]. Early reports from China drew the community attention on a higher risk of SARS-CoV-2 infection and related disease in cancer patients, whose outcomes appeared particularly unfavorable [20]. According to the most updated report from the Italian National Health Institute available at the time of writing, patients with active cancer within the last 5 years represented the 16% (419/2621) of those deceased at Italian hospitals and for whom data from a detailed chart review were available [21]. The debate concerning the risk of SARS-CoV-2 infection and related disease in cancer patients has rapidly gained a prominent place in the international scientific scenario. Clarifying the extent to which such risk may be affected by intrinsic disease characteristics, patients’ comorbidities, physiological features (e.g., age and gender) and treatment administered has become increasingly relevant in cancer patients’ management. As a result, in the process of decision making for therapeutic assignment, the oncologic community has been increasingly challenged by the need of evaluating the pros and cons of any therapeutic intervention against the risk of SARS-Cov-2 infection and disease [22].

The National Health Service classified cancer patients currently in treatment with chemotherapy, radiation therapy, targeted therapies (thyrosin-kinase, PARP inhibitors) or immunotherapy as subjects at ‘an utmost risk of infection’, in the specific guidelines that were released for the management of cancer patients during the Covid-19 pandemic. Patients that resulted SARS-CoV-2-positive while receiving ICIs were indicated as peculiarly susceptible for a severe form of infection [23]. In these regards, we join M Bersanelli in underlying the need of a separate discussion concerning the effects of ICIs in cancer patients with solid tumors compared with more ‘classical’ therapeutic approaches [24]. In such an attempt, our focus will be specifically placed on ICIs use in breast cancer.

Several discussion points may be raised. At first, in mTNBC, atezolizumab is administered in combination with chemotherapy. The two components of this combined regimen may both alter bone marrow functions and contribute to expose these patients to a higher risk of infections. However, the hematologic side effects due to nab-paclitaxel and atezolizumab differ significantly by of rates of occurrence and underlying mechanisms. Indeed, chemotherapy-induced neutropenia is mediated by the suppressive action exerted by chemotherapy on bone marrow myeloproliferative cells, with about up to a third of these patients developing febrile neutropenia. Conversely, the occurrence of hematological side effects caused by the ICIs is rare and mainly confined to autoimmune neutropenia and thrombocytopenia, hemolytic anemia and purpura. The pathogenetic mechanisms underlying immune-related adverse events are sustained by the cross-talk between immune-response and self-tolerance. Anti-PD-1 or anti-PD-L1 agents are not to be considered per se associated with immunosuppression [25–27]. In addition, severe neutropenia is barely consequent to ICIs use, with febrile neutropenia being even less frequent. In most of these cases, the ICI used is the anti-CTLA4 agent ipilimumab. In a subset patients treated with ICIs, ir-neutropenia has been documented and treated with prolonged immunosuppressive agents, including corticosteroids, whose use may be associated with an increased risk of infections and deserves close monitoring [28–30]. Data tend to be sparse concerning bacterial infections such as pneumonia or colitis, aspergillosis, pneumocystis pneumonia, infections by *Clostridium difficile*, or viral infections and reactivation in course of ICIs use [31–33]. In addition, data from a French Register (REISAMIC) on side effects of immunologic agents in cancer suggest that the use of anti-PD-1/PD-L1 may be related to the reactivation of tuberculosis, as observed in two patients from this register and three from the literature. The relative incidence of tuberculosis among patients receiving anti-PD-1/PD-L1 is about 1/1000 patients in France, and PD-1/PD-L1 pathways seem to be involved in tuberculosis infection pathophysiology by boosting tuberculosis specific T cells in patients with latent infections [34].

Second, lung toxicity in terms of interstitial pneumonia is a severe side effect, occurring in 2–5% of patients treated with anti-PD-1/PD-L1 and accounting for 35% of treatment-related toxic death [35]. There is a potential risk of overlapping between the drug-related side effects and an incipient form of Covid-19. The differential diagnosis of anti-PD-1 induced pneumonitis may complicate the management of patients receiving ICIs and suspected for Covid-19 [36]. In third place, the hypothesis of a role of anti-PD-1/PD-L1 agents in the pathogenesis of Covid-19 infection cannot be neglected. Indeed, the phenomenon renown as ‘cytokine-release syndrome’ (CRS), clearly outlined in its traits over the course of anti-PD-1/PD-L1 treatment, is characterized by high levels of IL-6 and other cytokines [37]. This phenomenon is also described in the late phase of Covid-19 infection, preluding to respiratory distress syndrome and multiple organ failure [38]. In Covid-19 patients, a decrease of peripheral CD4/CD8 T cells has been described, but their status is clearly hyperactivated, and an abundant interstitial mononuclear inflammatory infiltrate can be found in the lungs, mostly lymphocytes, which confirms the immune hyperactivation in the advanced stages of the infection [39]. Therefore, the hypothesis of a possible synergy between Covid-19 pathogenesis and anti-PD1/PD-L1 mechanism of action cannot be excluded. Some clues may help orient in the differential diagnosis. Cytokine storm is generally a late event in the SARS-CoV-2 infection and related disease, which generally follows disease diagnosis and oncologic treatment discontinuation. In addition, CRS occurrence seems quite uncommon in the case of anti-PD-1/PD-L1 use in breast cancer. Conversely, it is more commonly observed in lung cancer or melanoma, with the related estimates raising up to 10% of the patients treated. The ICIs agents more commonly associated with CRS belong to the anti-CTLA4 class. Otherwise, CRS occurrence follows the use of a combination of anti-CTLA4/anti-PD-1 therapy, and is often described in patients with a pre-existing lung/respiratory disease [40].

As a consequence of the Covid-19 pandemic, epidemiologic projections based on estimates from England foresee a 20% increase in cancer mortality, mainly due to delays in diagnosis and/or treatment administration [41,42]. Metastatic TNBC is a particularly aggressive breast cancer subtype, with extremely poor outcomes. In this patients subset, the administration of an effective treatment at the earliest time possible represents an imperative need. Unfortunately, at the time of writing, the effective therapeutic options available to this patients subsets are limited. The *a priori* exclusion of ICIs use in these patients under the shadow of a hypothetical reduction of risk of SARS-CoV-2 infection and disease could deprive them from the most active treatment currently available as first-line therapy. Data from real-world experiences are urgently needed to either sustain or disconfirm the aforementioned hypothesis. Meanwhile, systematic testing for ascertainment of Covid-19 status at the beginning of treatment and while on therapy appears necessary and functional to build a solid strategy in the short course, which may be implemented and inform future therapeutic decisions concerning ICIs use in mTNBC patients during and beyond the Covid era.
